# Public numbers on monetary valuation of fish landings

**DOI:** 10.1016/j.dib.2018.01.001

**Published:** 2018-01-06

**Authors:** Pedro Goulart, Francisco J. Veiga, Catarina Grilo

**Affiliations:** a Universidade de Lisboa, CAPP-ISCSP, Lisboa, Portugal; b Rua Almerindo Lessa, 1300-663 Lisboa, Portugal; c Universidade do Minho, NIPE-EEG, Braga, Portugal; d Calouste Gulbenkian Foundation, Lisbon, Portugal

**Keywords:** Fisheries, Economics, Prices, Revenue, Exchange rate, Portugal

## Abstract

This article presents a dataset that combines several time series of economic variables for Portugal, from 1969 to 2015, which can be used to accurately measure the change in value of different series in the fisheries sector. Raw data includes total nominal revenue from fish landings measured in thousands of euros, a consumer price index, and the nominal exchange rate of the Portuguese escudo against the US dollar (only until 1998). We use these raw data series to correct for inflation and to produce information on fish landings measured in constant prices in euros and to discuss the impact of the exchange rate depreciation in the fish sector if measured in US dollars. Data was retrieved from publicly accessible sources such as Statistics Portugal (*Instituto Nacional de Estatística*, INE) for fish landing revenue, AMECO (European Commission) for Consumer Price Index data, and International Financial Statistics (International Monetary Fund) for exchange rate data. The dataset is useful to academics, policymakers and advocacy groups alike for understanding the real production of the fisheries sector.

**Specifications Table**

**Table:** 

Subject area	*Environmental Sciences*
More specific subject area	*Fisheries Economics; International Economics; Public Economics*
Type of data	*Graph, figure*
How data was acquired	*Raw data was retrieved from publicly available databases (INE, AMECO, and IFS-IMF)*
Data format	*Raw data, estimated series*
Experimental factors	*N/a*
Experimental features	*N/a*
Data source location	*Portugal*
Data accessibility	*Data is available with this article*
Related research article	P. Goulart, F.J. Veiga, C. Grilo
	**The evolution of fisheries in Portugal: a methodological reappraisal with insights from economics**
	Fisheries Research, 199 (2018), pp. 76–80.

**Value of the Data**

• The raw data to construct the dataset comes from publicly available, reputable and leading sources such as the Portuguese statistics agency, a supranational entity as the European Commission, and an international organization such as IMF;
• The dataset describes the valuation of fish landings in current (nominal) and constant (real) prices valued in local currency (euros) to account for inflation and to estimate its importance in a country/region.
• The dataset describes the valuation of fish landings in foreign currency (nominal US dollars) to show the influence of the exchange rate.
• The dataset is useful to inform research and policymaking regarding the real production of the fisheries sector.

## 1 Data

The dataset is constructed from three raw series for Portugal. Data series includes fish landings measured in quantity, a consumer price index, and exchange rate data. Raw data was retrieved from publicly accessible sources such as Statistics Portugal (*Instituto Nacional de Estatística*, INE) for fish landings revenue data, AMECO (European Commission) for Consumer Price Index data, and International Financial Statistics (International Monetary Fund) for exchange rate data. The choice of the source also reflected on the leading role of each organization for that specific field.

### 1.1 Fish landings revenue data

Fish landings revenue data was retrieved from Statistics Portugal (*Instituto Nacional de Estatística*, INE). INE is responsible for directly collecting the data on Portugal or by collecting the information from a specific governmental body. For example, fish statistics available at INE are provided by DGRM, the national fisheries management authority, which receives official data from Docapesca, the state-run company that manages landing sites facilities and the first sale system in Portugal's mainland.

### 1.2 Consumer Price Index data

Consumer Price Index data for Portugal was retrieved from the AMECO database, the “annual macro-economic database of the European Commission's Directorate General for Economic and Financial Affairs” (as in its website). AMECO is the gold standard for European Commission analyses and publications. Consumer Price Index data is computed based on the evolution of prices in a basket of goods considered representative of the population's consumption. The original base year was 2005 and we have adjusted it for a 2015 base year – see the excel file.

### 1.3 Nominal exchange rate

Exchange rate data, on Portuguese escudos per US dollar – until 1998, and on Euros per US dollar – from 1999, was retrieved from the International Financial Statistics (IFS) of the International Monetary Fund (IMF). IMF has been the international institution created “to ensure the stability of the international monetary system” (as in its website) since 1944 following the Bretton Woods institutional framework. Because of the central role of exchange rates in the monetary system, IMF's IFS is the leading database regarding this topic. The nominal exchange rate used here corresponds to the average during the respective year.

## 2 Experimental design, materials, and methods

From the retrieved fish landings revenue measured in current (nominal) prices (1), we use the available raw data series to produce information on fish landings measured (2) in constant (real) prices and (3) in current (nominal) US dollars. See [1] for further details.

### 2.1 Fish landings measured in current prices

**Fig. 1:**
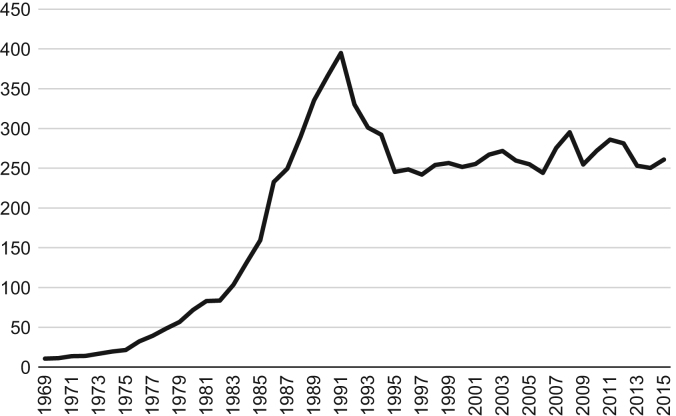
Fish landings measured in Euros, current prices, 1969–2015.Source: Statistics Portugal (INE).

Fig. 1 portrays revenue from fish landings in current prices (in Euro) for Portugal. The period covers the publicly available official statistics from 1969 to 2015. The series depicts an increasing trend until 1991, after which there is a decline. From 1991, fish landings decrease from almost 400 million to *circa* 250 million euros in 1995, followed by stabilization around this value, in spite of some fluctuation particularly after 2006.

### 2.2 Fish landings measured in constant prices

**Fig. 2:**
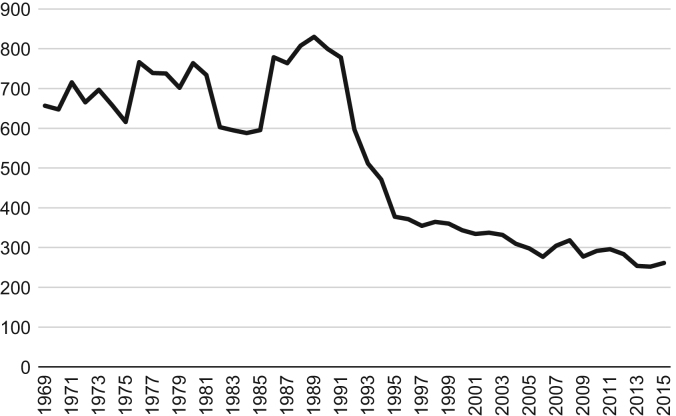
Fish landings measured in Euros, constant prices (of 2015), 1969–2015.Source: Authors’ calculations based on Statistics Portugal (INE) and AMECO (European Commission).

Fish landings measured in current prices (nominal revenues) do not account for the loss of purchasing power due to inflation. Revenue from fish landings measured in constant prices (real revenue) does account for inflation. The values from 1969 to 2015 are portrayed in Fig. 2. This type of correction is standard for longitudinal series measured in monetary units, such as Gross Domestic Product or wages (e.g. see figure 5 in [2]; Chapter 3 in [3]).

This series provides evidence of a mean fish landing value of around 700 million euro (valued at 2015 prices), but with an increasing variance from 1969 to 1989. The latter is the peak year, reaching almost 830 million euro. A very strong decline, from 1991 to 1995, halved the real value of fish landings to 380 million. Thereafter, there was a slower but consistent decline to 260 million euros in 2015.

While deflating series is necessary, the choice of an appropriate deflator is not obvious or easy. This issue has generally concerned academic [4] and policymaking [5] spheres and has led to alternative strategies and deflators. Long historical series are often scarce and alternative choices are rare. Particularly, using a general purpose deflator such as the CPI may have its limitations, depending on the purpose, but is widely used. When applying this approach to the fisheries sector, we obtain the purchasing power of fish revenues. An alternative way forward is to measure the value of fish production itself by extending backwards the FAO's Fish Price Index available from 1990 onwards and use it to deflate the current price series [6].

### 2.3 Fish landings measured in current US Dollars

For international comparisons, revenue series should be valued in a common currency. The traditional approach is to convert the revenue series of different countries to a currency of general use, such as the US Dollar. The nominal exchange rate of the Portuguese Escudo against the US Dollar (PTE/USD) is used for the period until 1998. We first convert the euro amounts to Portuguese Escudos using the conversion rate of 200.482 PTE per Euro. Then, we convert the PTE to dollars using the PTE/USD exchange rate. For the period from 1999 onwards we simply convert the Euro amounts to USD using the nominal Euro/USD exchange rate.

**Fig. 3:**
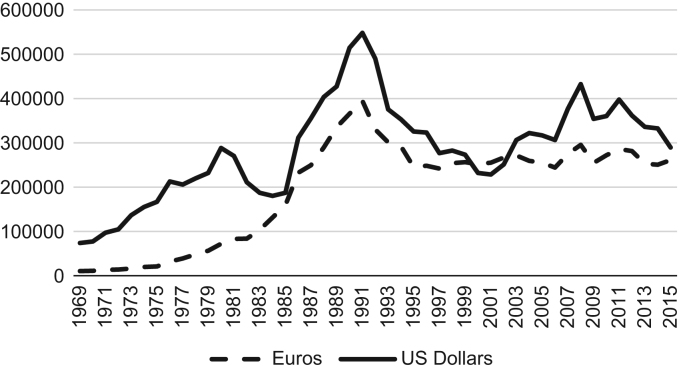
Fish landings measured in US Dollars and Euros, current prices, 1969–2015.Source: Authors’ calculation based on Statistics Portugal (INE) and IFS (IMF).

Fig. 3 presents the revenue from fish landings measured in current US Dollars, with the values in current Euros in the background. The effect of the nominal exchange rate is visible, for example, in the period from 1980 to 1985, during which there was a strong depreciation of the Portuguese Escudo. In this period, the growth trends of the two series clearly diverged. For Portugal, a large net importer of fish, the exchange rate is particularly important when considering the value of fish imports.

## Funding sources

The authors would like to thank the FCT/MEC's financial support through national funding and by the ERDF through the Operational Programme on Competitiveness and Internationalization – COMPETE 2020 under the PT2020 Partnership Agreement, to Francisco Veiga [references FCOMP-01–0124-FEDER-037268; [POCI-01-0145-FEDER-006683 and UID/ECO/03182/2013 and to Pedro Goulart [reference UID/CPO/00713/2013 PEst-OE/CJP/UI0713/2013].
